# Supplementary material for: Structural dynamics of a metal–organic framework induced by CO2 migration in its non-uniform porous structure
Source: Nat Commun. 2019 Mar 1;10:999. doi: 10.1038/s41467-019-08939-y (PMC6397191; doi:10.1038/s41467-019-08939-y)
Supplement: Supplementary file 1 — Supplementary Information [file 41467_2019_8939_MOESM1_ESM.pdf]

Supplementary Information for

Structural dynamics of a metal-organic framework induced by CO_2 migration in its non-uniform porous structure

Zhao et al.

Supplementary Methods

1. Synthesis

ZIF-7 was synthesized using a solvothermal method. 0.75 g of zinc nitrate hexahydrate ($Zn(NO_3)_2 \cdot 6H_2O$, 2.52 mmol) and 0.25 g of benzimidazole (HbIm, $C_7H_6N_2$, 2.05 mmol) were first dissolved in 75 ml of fresh dimethylformamide (DMF). The resultant solution was then sealed into a 100 ml Teflon-lined stainless steel autoclave. The autoclave was heated at 400 K for 48 h. After naturally cooling to room temperature, white platy crystals were isolated after the mother liquor was removed. The solid product was washed thoroughly with methanol. The average yield was around 0.34 g (97% based on HbIm). All chemicals employed were commercially available (Sigma-Aldrich and Acros Organics), with purity of 98% or above, and were used as received.

2. Laboratory characterization

The crystal structures of ZIF-7 samples were characterized by X-ray powder diffraction (XRD) using a Bruker D8 Advance X-ray diffractometer operated at 40 kV and 40 mA with Cu Kα_1 radiation (λ = 1.54056 Å). The XRD data of the as-synthesized sample were compared with that calculated from the ZIF-7-I crystallographic model in the literature[1]. ZIF-7-I purity was around 100%. The morphology of ZIF-7 samples was examined by scanning electron microscopy using a JEOL JSM-5510 microscope. CO_2 adsorption isotherms were measured by a Hiden Isochema IGA gravimetric analyzer. The as-synthesized sample was activated at 400 K in air for 48 h before each measurement. Before data collection, the sample was vacuumed *in situ* (~10^{-4} mbar) at room temperature for 3 h to remove any remaining trace guest molecules.

3. *In situ* synchrotron X-ray powder diffraction (SXRD)

In situ SXRD data were collected at beamline I11, Diamond Light Source, Harwell, UK. The energy of the incident X-ray was set at ~15 keV[2]. The wavelength (λ = 0.82634(1) Å) and the 2θ zero point were determined by fitting the diffraction data of a high-quality silicon powder (SRM640c).

The as-synthesized sample was first activated at 400 K in air for 24 h. The activated sample was then loaded into a quartz glass capillary (0.5 mm). Glass wool was packed on top of the sample and the capillary was then fixed onto a custom-made gas cell. Before data collection, the sample cell was evacuated to 10^{-5} Pa at room temperature for 3 h to remove any remaining trace guest molecules from the sample. CO_2 pressure was controlled by a custom-made gas loading system. After each CO_2 loading, 2 min was allowed before data collection for the system to reach equilibrium.

Since sample spinning was prohibited due to the position of the gas cell, the sample was rocked through ±10° about the ω-axis to improve powder averaging. SXRD data were collected with Mythen II position sensitive detectors (PSD) over the 2θ range of 2–90°, with two datasets offset by 0.25^o (2θ) collected (each for 30 s). These datasets were subsequently automatically merged to remove gaps in the data where the microstrips (X-ray detection modules) join. PSD and data treatment have been described in detail in the literature[3].

4. Quasi-elastic neutron scattering (QENS)

QENS data were collected using the IRIS indirect geometry time-of-flight (TOF) spectrometer at ISIS Neutron and Muon Source, Rutherford Appleton Laboratory, Harwell, UK. At IRIS, the sample first got a white beam of neutrons containing a band of energy. After scattering, the crystal analyzer (the (002) plane of pyrolithic graphite) chose a single final energy (1.84 meV) to send to detectors. Scattered neutrons were detected over an angular range of $2\theta = 25–160^o$. The instrumental resolution and detector efficiencies were calibrated by fitting the spectrum of a vanadium standard. The elastic energy resolution was 17.5 μeV. The energy transfer range was -0.4–0.4 meV and the Q range was 0.42–1.85 $Å^{-1}$. IRIS was also built with long-wavelength diffraction capability. The d range was 1–12 Å with $\Delta d/d = 2.5 \times 10^{-3}$.

The as-synthesized sample was first activated at 400 K in air for 24 h. The activated sample (*ca.* 4.6 g) was then wrapped into two pieces of aluminum foil to make a sample lining fitted into the annular space of an aluminum cylinder sample cell (Ø 24/28 mm × *h* 65.6 mm). The thickness of the sample lining was about 2 mm. Glass wool was placed in the top of the sample cell. The sample cell was then sealed with indium and connected to a custom-made gas loading system. During data collection, the temperature of the sample was controlled by a helium cryostat and thermocouples. QENS spectra were first recorded when the sample was under vacuum. At 15 and 225 K, the accumulated proton current was 600 μA for good statistics. At 298 K, the accumulated proton current was 1146.8 μA. CO_2 was then loaded into the sample cell at 298 K to enable ZIF-7 to reach a CO_2 uptake of 1.3 $mmol \cdot g^{-1}$. For QENS data of the loaded sample collected at 15, 225 and 298 K, the accumulated proton current was 600 μA. Diffraction data were collected simultaneously with QENS data collection.

CO_2 loading was measured using the method employed in the literature[4-6]. A gas handling system shown in Supplementary Fig. 3 was used for gas loading. The volume of the system without the sample cell (i.e. to the valve on top of the cell) ($V1$) was measured using N_2. A certain amount of N_2 ($n0$) was loaded into the vacuumed system at room temperature so that a pressure of $p0$ was reached (shown on the barometer). According to the ideal gas law, we can obtain the value of $V1$:

$$p0V1 = n0\mathrm{R}T \qquad \text{Supplementary Equation 1}$$

The volume of the system including the sample cell ($V2$) was also measured using the same method. ZIF-7 has almost no N_2 adsorption capacity at 298 K[7], so $V2$ is the actual volume of the system. After the sample was evacuated to 10^{-5} Pa at room temperature for 16 h to remove any remaining trace guest molecules, the valve on top of the sample cell was closed. CO_2 was then loaded into the system to reach a pressure of $p1$. The amount of CO_2 in the system ($n1$) can be calculated:

$$p1V1 = n1\mathrm{R}T \qquad \text{Supplementary Equation 2}$$

Then the valve on top of the sample cell was open, CO_2 pressure will stabilize at $p2$. The amount of CO_2 left in the system ($n2$) can also be calculated:

$$p2V2 = n2\mathrm{RT} \qquad \text{Supplementary Equation 3}$$

The amount of CO_2 adsorbed (n) by ZIF-7 is:

$n = n1 - n2$ Supplementary Equation 4

As we know the amount of sample used in each experiment (*ca.* 4.6 g), we can precisely control the CO_2 loading to be 1.3 $mmol\cdot g^{-1}$.

5. Inelastic neutron scattering (INS)

INS data were collected using the TOSCA TOF spectrometer at ISIS Neutron and Muon Source, Rutherford Appleton Laboratory, Harwell, UK. TOSCA has a wide energy transfer range of -2.5–1000 meV (-20–8050 cm^{-1}) and a high energy resolution of *ca.* 1.25% $\Delta E/E$.

The sample environment at TOSCA was similar to that at IRIS. Before data collection, the as-synthesized sample was first activated at 400 K in air for 24 h. The activated sample (*ca.* 4.6 g) was then wrapped into two pieces of aluminum foil to make a sample lining for a stainless steel cylinder sample cell (Ø 16 mm × *h* 76 mm). Glass wool was placed in the top of the sample cell to prevent any sample spillage during the experiment. The sample cell was then sealed with a copper O-ring and connected to a custom-made gas loading system. The sample chamber was evacuated to 10^{-5} Pa at room temperature for 16 h to remove any remaining trace guest molecules from the sample. During data collection, the temperature of the sample was controlled by a closed cycle refrigerator (CCR) and was kept below 10 K to minimize the thermal motion of CO_2 molecules and the host framework which influences the accuracy of the measurement. Namely, at higher temperatures the increase in Debye-Waller factor causes broadening of the spectral peaks, i.e. decrease in peak intensity or spectral resolution. INS spectra were first recorded when the sample was under vacuum. The accumulated proton current was 3604 μA for good statistics. CO_2 was then loaded into the sample cell at 298 K to enable ZIF-7 to reach a CO_2 uptake of 1.3 $mmol\cdot g^{-1}$. CO_2 loading was measured using the same method as that in the QENS experiment. During data collection of the loaded sample, the accumulated proton current was 3558.6 μA.

6. Density functional theory (DFT) calculation

Models for the DFT calculations were built based on the crystal structures from the literature[8,9]. One ZIF-7 unit cell was used with a chemical formula of $Zn_{18}C_{252}N_{72}H_{180}$. Perdew-Burke-Ernzerhof (PBE) generalized gradient approximation (GGA)[10] implemented in the VASP package[11] was used. The projector augmented wave (PAW)[12] pseudopotential method was employed. Due to the large unit cell of ZIF-7, only Gamma point was sampled. The cutoff energy was 400 eV. The shape of the unit cell and atomic positions were allowed fully relaxed during the optimization. The energy convergence for the self-consistent electronic relaxation was set to be 10^{-6} eV and the force convergence was set to be 0.01 $eV\cdot Å^{-1}$. To count in the weak dispersive interaction, van der Waals interactions (as implemented in the DFT-D2 scheme[13,14]) were considered during the calculations. We included a typical input file of our DFT calculations as a supporting file (Supplementary Data 1). Details of the calculation can be found in this file.

7. Grand canonical Monte Carlo (GCMC) molecular simulations

CO_2 adsorption isotherms in ZIF-7 were investigated using grand canonical Monte Carlo (GCMC) simulations performed in the multi-purpose code RASPA[15]. ZIF-7-I and ZIF-7-II models were built based on the crystal structures from the literature[8,9]. Framework atoms were kept fixed at the crystallographic positions. In order to correctly describe the absence of CO_2 molecules in pores A before the ZIF-7-II to ZIF-7-I phase transition, pores A were all blocked in ZIF-7-II during the simulation. We used the standard Lennard-Jones (LJ) 12-6 potential to model the interactions between the framework and fluid atoms. In addition, a Coulomb potential was used for fluid-fluid interactions. The parameters for framework atoms were derived from the Universal Force Field[16] and those previously developed for ZIF-8[17]. CO_2 molecules were modeled using the TraPPE potential with charges placed on each atom and at the center of mass (Supplementary Table 5)[18]. EQeq was used to assign the partial charges of the framework. The Lorentz-Berthelot mixing rules were employed to calculate fluid-solid LJ parameters, and LJ interactions beyond 12.8 Å were neglected. The Ewald summations method was used to compute the electrostatic interactions. Up to 50,000 Monte Carlo cycles were performed, the first 50% of which were used for equilibration, and the remaining steps were used to calculate the ensemble averages. Monte Carlo moves consisted of insertions, deletions, displacements, and rotations. In a cycle, N Monte Carlo moves are attempted, where N is defined as the maximum of 20 or the number of adsorbates in the system. To calculate the gas-phase fugacity we used the Peng-Robinson equation of state[19]. The isosteric heat of adsorption (Q_{st}) was calculates using the fluctuation theory[20]. Input files are included in Supplementary Data 2.

Supplementary Discussion

1. QENS data analysis

TOF data were converted to energy transfer using the Mantid software[21]. In each measurement, 50 QENS spectra were recorded over the energy transfer range (-0.4–0.4 meV, 0.42–1.85 Å^{-1} in Q). To fit each QENS spectrum, a delta function and a Lorentzian function were used to describe the elastic and coherent quasi-elastic scattering from the sample, respectively (Supplementary Fig. 4). They were convolved by the scattering function of the vanadium standard, which is close to a Lorentzian function. The baseline was regarded as a straight line. Sequential fitting was conducted. The QENS spectrum collected at Q = 0.44 Å^{-1} was first manually fitted. The starting functions used for fitting each following spectrum at Q = 0.48–1.85 Å^{-1} were the calculated results from the previous spectrum in the sequence.

No coherent quasi-elastic scattering signal can be found in the QENS spectra of ZIF-7 under vacuum. This indicates that the dynamics of ZIF-7 do not contribute to the QENS spectra of CO_2-loaded ZIF-7, regardless of the ZIF-7-II to ZIF-7-I phase transition observed by simultaneous neutron powder diffraction. No coherent quasi-elastic scattering signal can be found in the QENS spectra of CO_2-loaded ZIF-7 at 15 K. This suggests that CO_2 was frozen in ZIF-7 at 15 K.

Thus the coherent quasi-elastic scattering signal in the QENS spectra of CO_2-loaded ZIF-7 at 225 and 298 K predominantly reflects the collective motion of CO_2 molecules in ZIF-7 at these temperatures. The contribution from the vibrational and rotational motions of CO_2 molecules is negligible: the vibrational motion only affects the measured intensity through a Debye-Waller factor; for a linear and symmetrical molecule like CO_2, the influence of the rotational motion is very small in the QENS domain[22]. We can say that the coherent quasi-elastic scattering signal in the QENS spectra of CO_2-loaded ZIF-7 at 225 and 298 K predominantly reflects the collective translational motion of CO_2 molecules, i.e. CO_2 transport diffusion at these temperatures.

Over a small Q range (i.e. large length scales), assuming CO_2 transport diffusion is isotropic, the coherent quasi-elastic scattering function has the form[22]:

$$S_{Coh}(Q,\omega) = \frac{S(Q)}{\pi} \times \frac{D(Q)Q^2}{\omega^2 + (D(Q)Q^2)^2} = S(Q)L \qquad \text{Supplementary Equation 5}$$

where $D(Q)$ is CO_2 transport diffusion coefficient and L corresponds to a Lorentzian function. The half-width at half-maximum (HWHM) of L is the product of $D(Q)$ and Q^2:

$$HWHM = D(Q)Q^2 \qquad \text{Supplementary Equation 6}$$

where HWHM is in s^{-1}, Q is in m^{-1} and D_t is in m^2·s^{-1}. At larger Q values (*i.e.* over smaller length scales), the relationship between HWHM and Q^2 is no longer linear due to CO_2 elementary jumps between adsorption sites (Supplementary Fig. 5). Since we are simply interested in CO_2 long-range diffusion (i.e. small Q range), the calculation of $D(Q)$ can be based on Supplementary Equation 6. Fitting the QENS spectra of CO_2-loaded ZIF-7 at 225 and 298 K allows the calculation of $D(Q)$ over the whole Q range. By fitting $D(Q)$ with a polynomial function, $D(0)$, i.e. macroscopic CO_2 transport diffusivity D_t, can be

determined[22]. CO_2 transport diffusivity in zeolites usually ranges from 10^{-10} to 10^{-8} $m^2 \cdot s^{-1}$ [23,24]. CO_2 transport diffusivities in ZIF-7 at 225 and 298 K (6.3(7) × 10^{-9} $m^2 \cdot s^{-1}$ and 4(2) × 10^{-11} $m^2 \cdot s^{-1}$, respectively) are comparable with those in zeolites.

While transport diffusion reflects the collective motion of CO_2 molecules caused by local concentration gradients, self-diffusion often describes the displacement of individual CO_2 molecules, i.e. elementary jumps between adsorption sites. In zeolites, D_t has a close relationship with self-diffusivity D_s [22]:

$$D_t = D_s\left(\frac{\partial \ln p}{\partial \ln c}\right) \quad \text{Supplementary Equation 7}$$

where $\partial \ln p / \partial \ln c$ is the thermodynamic correction factor and can be calculated directly from the CO_2 adsorption isotherm. p is CO_2 pressure in kPa and c is CO_2 uptake at p in mmol·g^{-1}. Considering the similarity between zeolites and ZIFs (zeolitic imidazolate frameworks), CO_2 self-diffusivities in ZIF-7 at 225 and 298 K can be calculated based on Supplementary Equation 7. At CO_2 loading = 1.3 mmol·g^{-1} and T = 195 K, $\partial \ln p/\partial \ln c$ = 2.6 from Fig. 1a. Assuming $\partial \ln p/\partial \ln c$ is still 2.6 at 225 K, considering D_t = 4(2) × 10^{-11} $m^2 \cdot s^{-1}$, D_s = 1.5(8) × 10^{-11} $m^2 \cdot s^{-1}$. At CO_2 loading = 1.3 mmol·g^{-1} and T = 298 K, $\partial \ln p/\partial \ln c$ = 33.3 from Fig. 1a and D_t = 6.3(7) × 10^{-9} $m^2 \cdot s^{-1}$, thus D_s = 1.9(2) × 10^{-10} $m^2 \cdot s^{-1}$.

2. INS data analysis

The INS spectrum of ZIF-7 at a CO_2 loading of 1.3 mmol·g^{-1} was compared with that of ZIF-7 without CO_2 loaded (ZIF-7-II) (Supplementary Fig. 7A). Peaks were assigned based on previous works[25-27] (Supplementary Table 4). In the ZIF-7-II spectrum, a doublet at 230–245 cm^{-1} represents the out-of-plane bending motion of bIm linkers at the bridge of the imidazole ring (Im) and the benzene ring (Bz) (Supplementary Fig. 7B). The ratio between peak areas at 242 cm^{-1} and 234 cm^{-1} is 1:2.3. As shown in Fig. 1d, linkers in ZIF-7-II are distributed in two types of pore B. The ratio between the number of type I and II pore B is 1:2, thus the INS peak at 242 cm^{-1} corresponds to the out-of-plane bending motion of the linkers in type I pore B. After CO_2 loading, the 242 cm^{-1} peak merges into the 234 cm^{-1} peak, this peak shift matches well with what is expected when 0.5 CO_2 molecule is adsorbed per linker in type I pore B, as predicted by neutron powder diffraction results[9]. According to Hooke's Law,

$$\varpi \propto \sqrt{\frac{k}{m}}$$

Supplementary Equation 8

where ω is wavenumber, k is the elastic constant, m is mass. Thus,

$$\frac{\varpi_0}{\varpi_f} = \sqrt{\frac{m_0 + N\Delta m}{m_0}}$$

Supplementary Equation 9

where ω_0 is the wavenumber when the linker is without CO_2 adsorbed, ω_f is the wavenumber when the linker is adsorbed with CO_2, m_0 is the mass of one bIm linker, Δm is mass of one CO_2 molecule, N is the number of CO_2 molecules adsorbed per linker. When N = 0.5, ω_0 = 242 cm^{-1} and ω_f = 234 cm^{-1},

$$\sqrt{\frac{m_0 + N\Delta m}{m_0}} = 1.08 \approx \frac{\varpi_0}{\varpi_f}$$

Supplementary Equation 10

Thus we can conclude that the linkers of type I pore B are mainly responsible for the initial CO_2 adsorption, rather than those of type II pore B.

A higher-frequency peak occurs after CO_2 loading in the energy ranges corresponding to Im torsion (644 cm^{-1}) and the out-of-plane bending motion of Im C–H (843 cm^{-1}) (Supplementary Fig. 7D). The occurrence of higher-frequency peaks could be due to the formation of hydrogen bonds between CO_2 and Im C–H. The ratio between peak areas at two frequencies is approximately 1:1. This is because only half of the linkers can adsorb CO_2 on its Im (Supplementary Fig. 7E).

Admittedly, ZIF-7-II to ZIF-7-I phase transition can also induce peak shift and affect that induced by CO_2 adsorption. For the torsion motion of Bz (415–435 cm^{-1}, Supplementary Fig. 7C), the ratio between peak areas at 419 cm^{-1} and 429 cm^{-1} is 1:1.6. Bz in type I pore B is more relaxed since they are further away from each other when compared with those in type II pore B. After CO_2 loading, two peaks merge into one, indicating type I and II pore B became one structure (Fig. 1d). The narrowing of 898 cm^{-1} peak (the out-of-plane bending motion of Bz C–H) is also due to the same reason.

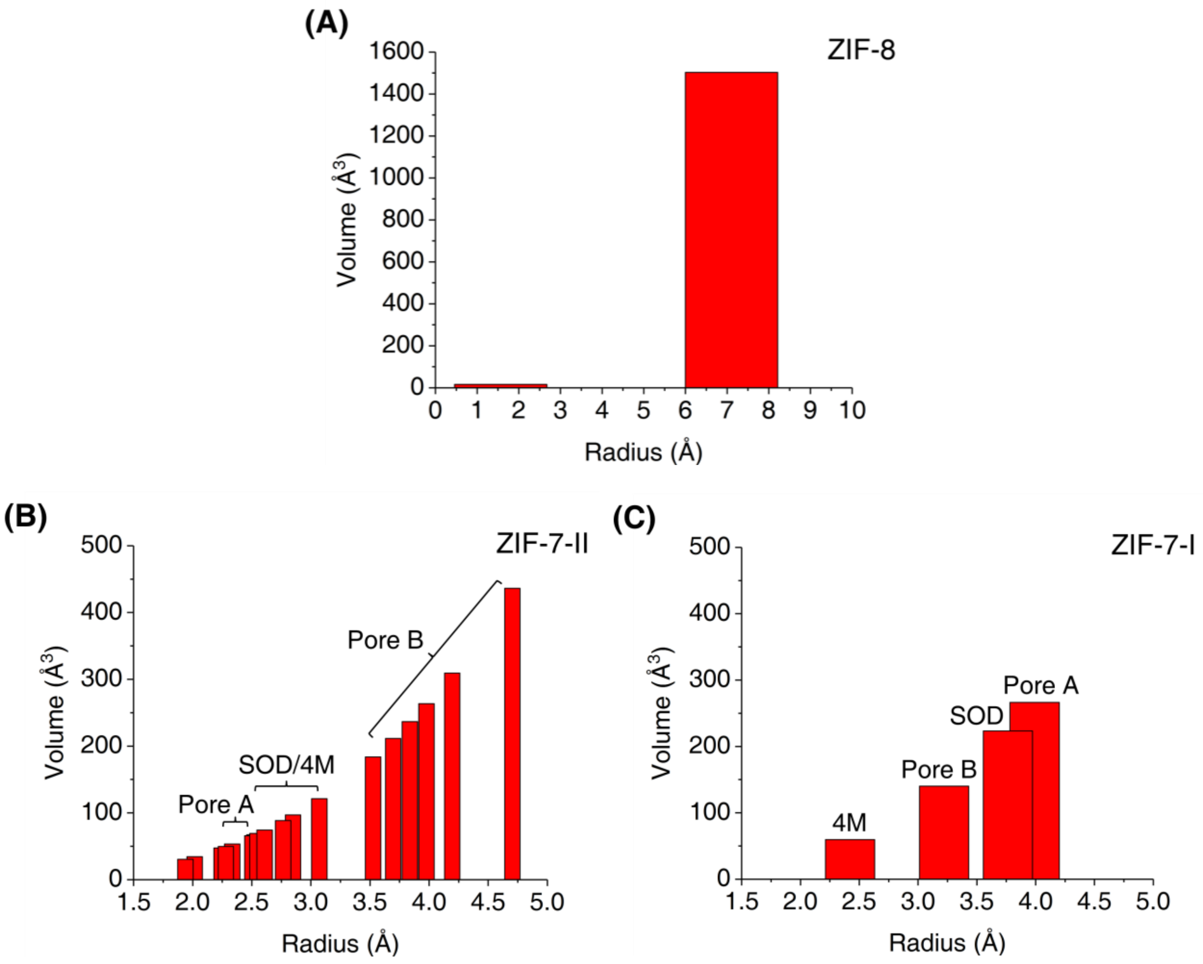

Supplementary Figure 1 Pore size distribution of ZIF-7-I, ZIF-7-II, and ZIF-8, measured by CrystalMaker 10.3 software using their crystal structures[1,8]. The probe radius was 1.55 Å, the same as the van der Waals radius of a N_2 molecule. SOD: sodalite cage, 4M: four-member-ring pore. Values of pore sizes are listed in Supplementary Table 1.

From the analysis results, it is clear that there is only one type of pores in ZIF-8 (7.1 Å), which corresponds to the sodalite cage of ZIF-8. This indicates that ZIF-8 has a uniform porous structure. In ZIF-7-I, four types of pores with distinct radius were found: pore A (4.0 Å), sodalite cage (3.8 Å), pore B (3.2 Å), and four-member-ring pore (2.4 Å). The pore distribution in ZIF-7-II is very different from that in ZIF-7-I. During ZIF-7-II to ZIF-7-I phase transition, the radius of pore B decrease from 3.5 – 4.7 Å to 3.2 Å, whereas the radii of pore A and sodalite cage increase from 2.3 – 2.5 Å and 2.8 – 3.1 Å to 4.0 Å and 3.8 Å, respectively. The radius of four-member-ring pore remains unchanged (2.5 – 2.9 Å to 2.4 Å).

As ZIF-7-II to ZIF-7-I phase transition is induced by CO_2 adsorption, it can be said that with the filling of CO_2, pore B decreases in size whereas pore A increases in size. This strengthens our proposed mechanism on the sequential CO_2 filling of pore B (first) and pore A (last). The filling of pore A with CO_2 requires pore deformation which is induced by the interaction between CO_2 and pore B.

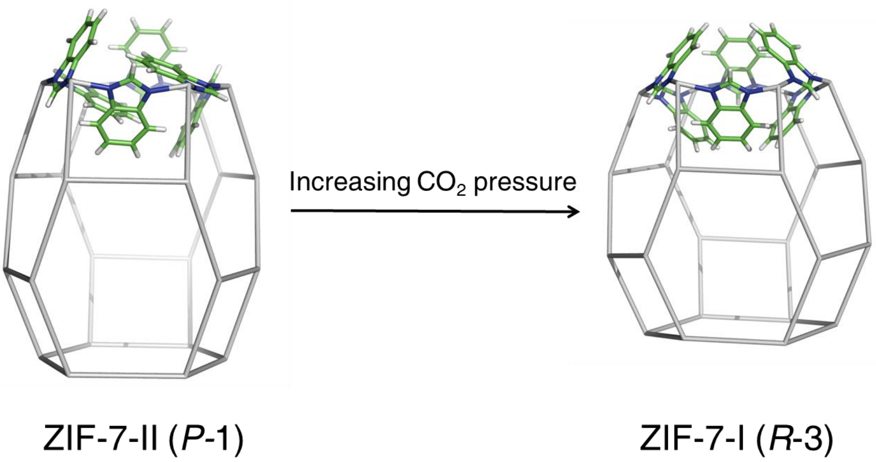

Supplementary Figure 2 The crystal structures of ZIF-7-I and ZIF-7-II. One pore A is shown. The other part of the framework is simplified by replacing Zn−bIm−Zn with Zn−Zn (Zn: grey).

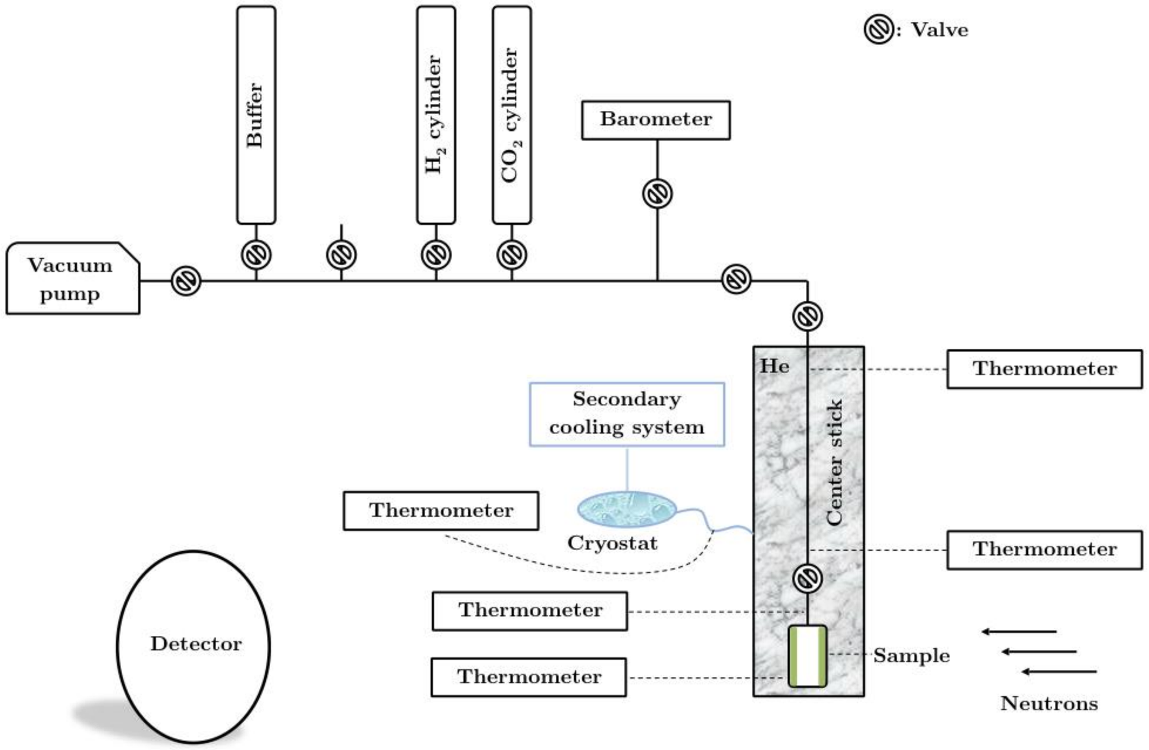

Supplementary Figure 3 A sketch of the setup of QENS and INS experiments.

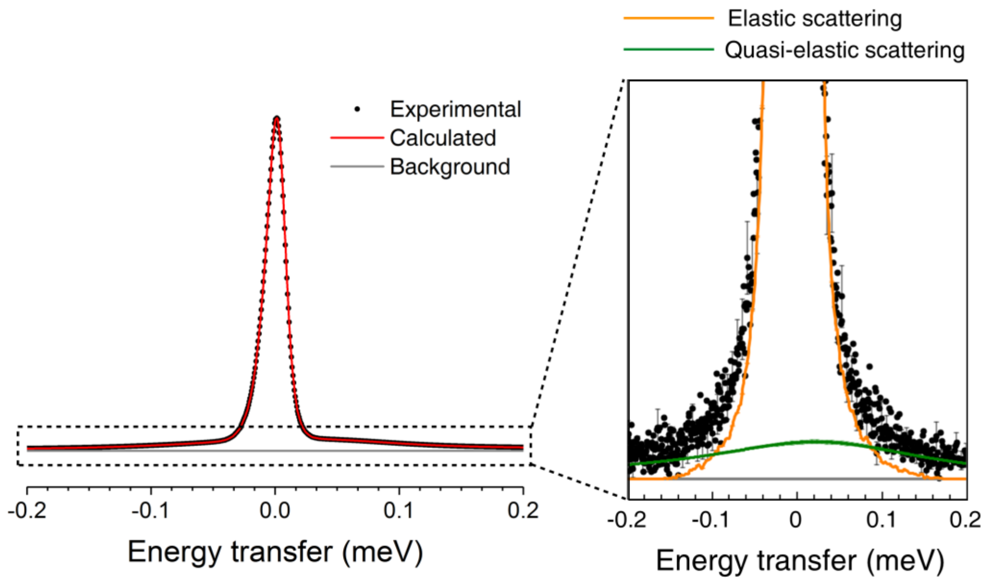

Supplementary Figure 4 A fitted QENS spectrum of CO_2-adsorbed ZIF-7. CO_2 loading = 1.3 mmol·g^{-1}, T = 298 K, Q = 0.44 Å^{-1}. The contribution from elastic and quasi-elastic scattering is illustrated in the enlarged figure on the right side.

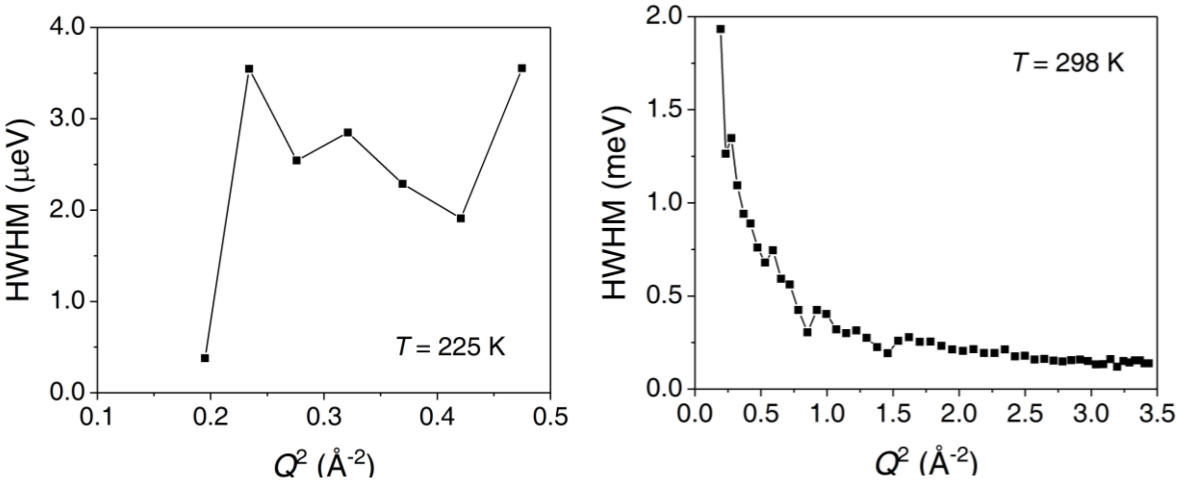

Supplementary Figure 5 The Q^2 dependence of the HWHM of the Lorentzian component of each QENS spectrum at 225 and 298 K. The non-linear dependence suggests non-Fickian diffusion.

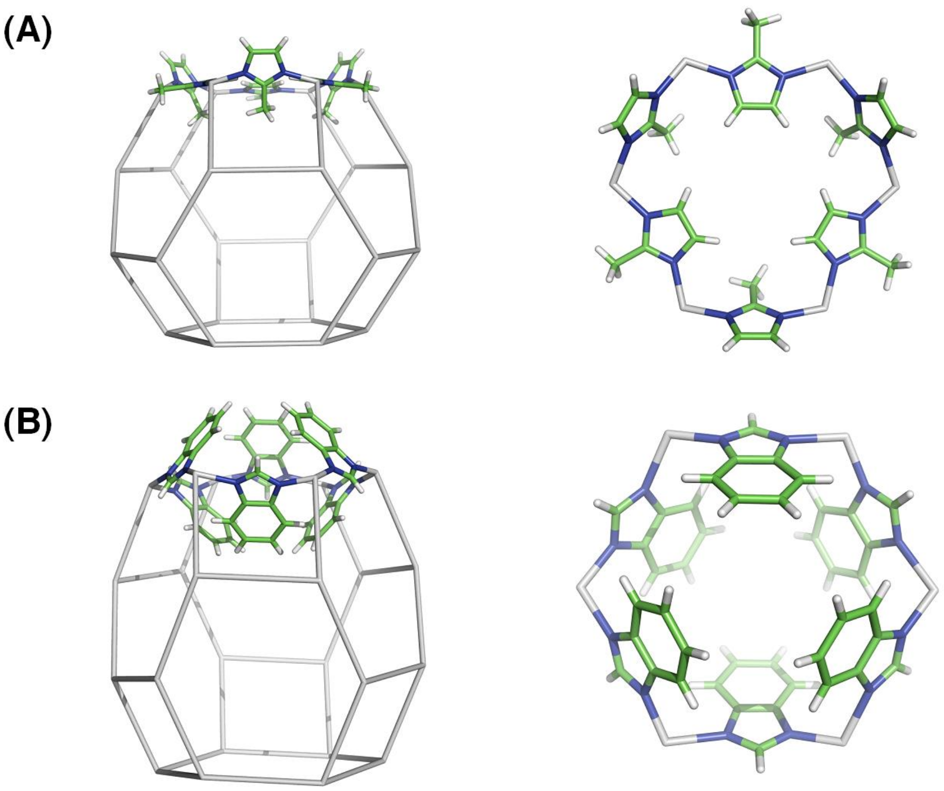

Supplementary Figure 6 The building unit, sodalite cage, of ZIF-8 (A) and ZIF-7 (B). The six-member-ring of ZIF-8 resembles the geometry of pore A in the ZIF-7. Its window size (diameter 3.4 – 4.1 Å) is similar to that of pore A (3 Å).

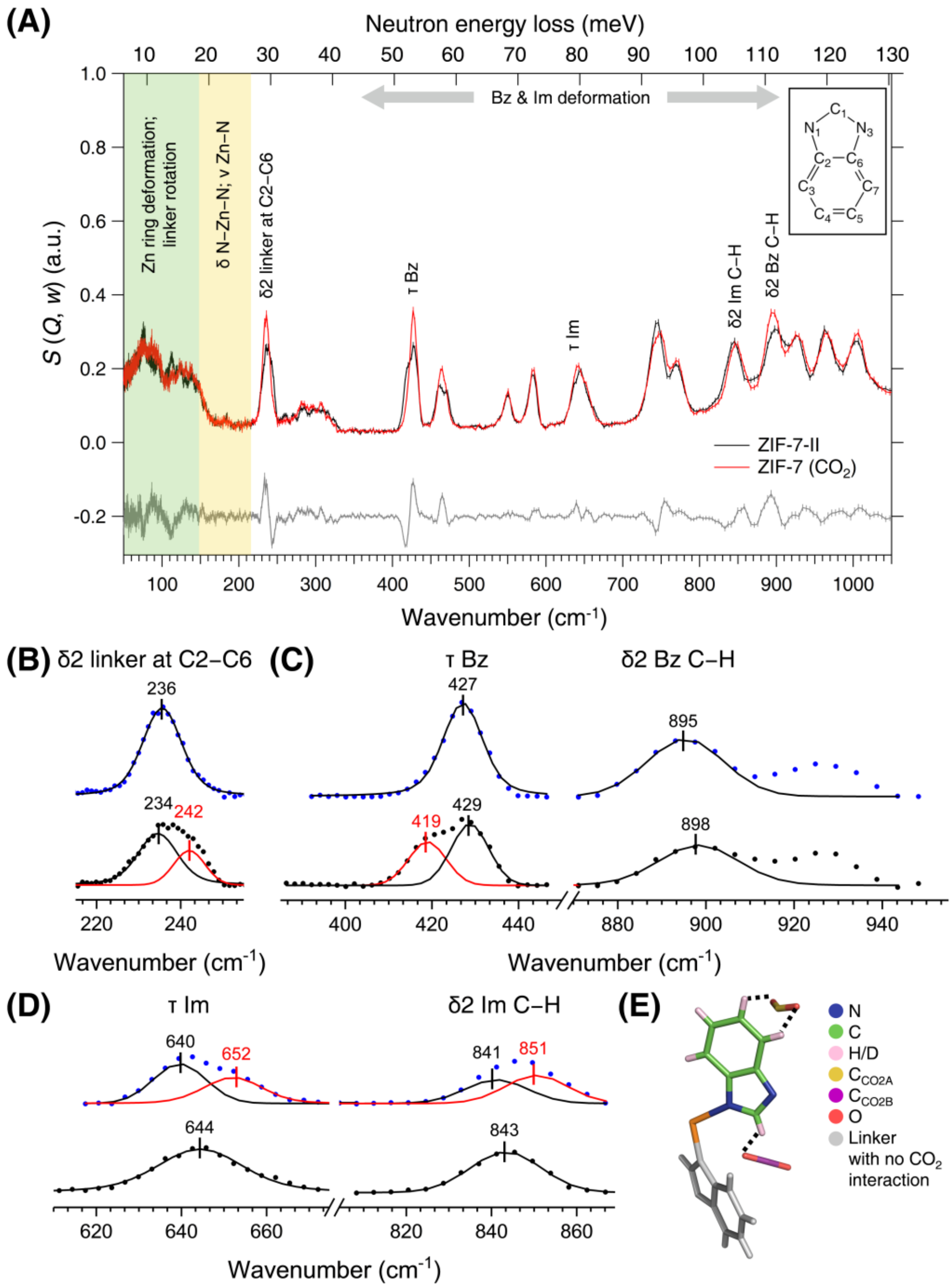

Supplementary Figure 7 (A) INS spectra of ZIF-7-II and CO_2-adsorbed ZIF-7. Difference between ZIF-7 (CO_2) and ZIF-7-II spectra is shown at the bottom of the figure: ZIF-7 (CO_2) spectrum subtracted by ZIF-7-II spectrum. ν: stretching; δ: bending (δ1: in-plane, δ2: out-of-plane); τ: torsion. Bz: benzene ring; Im: imidazole ring. The inset shows the atom nomenclature used for linker; H is omitted for clarity.1 meV ≡ 8.05 cm^{-1}. **(B–D)** Detailed INS spectra with peak(s) fitted. ZIF-7-II: black dots, CO_2-adsorbed ZIF-7: blue dots. **(E)** The asymmetric unit of CO_2-adsorbed ZIF-7 crystal structure. Linker: N, blue; C, green; H/D, pink. C of CO_2 in pore A: yellow, C of CO_2 in pore B: purple, O: red. The linker not interacting with CO_2 is shown in grey.

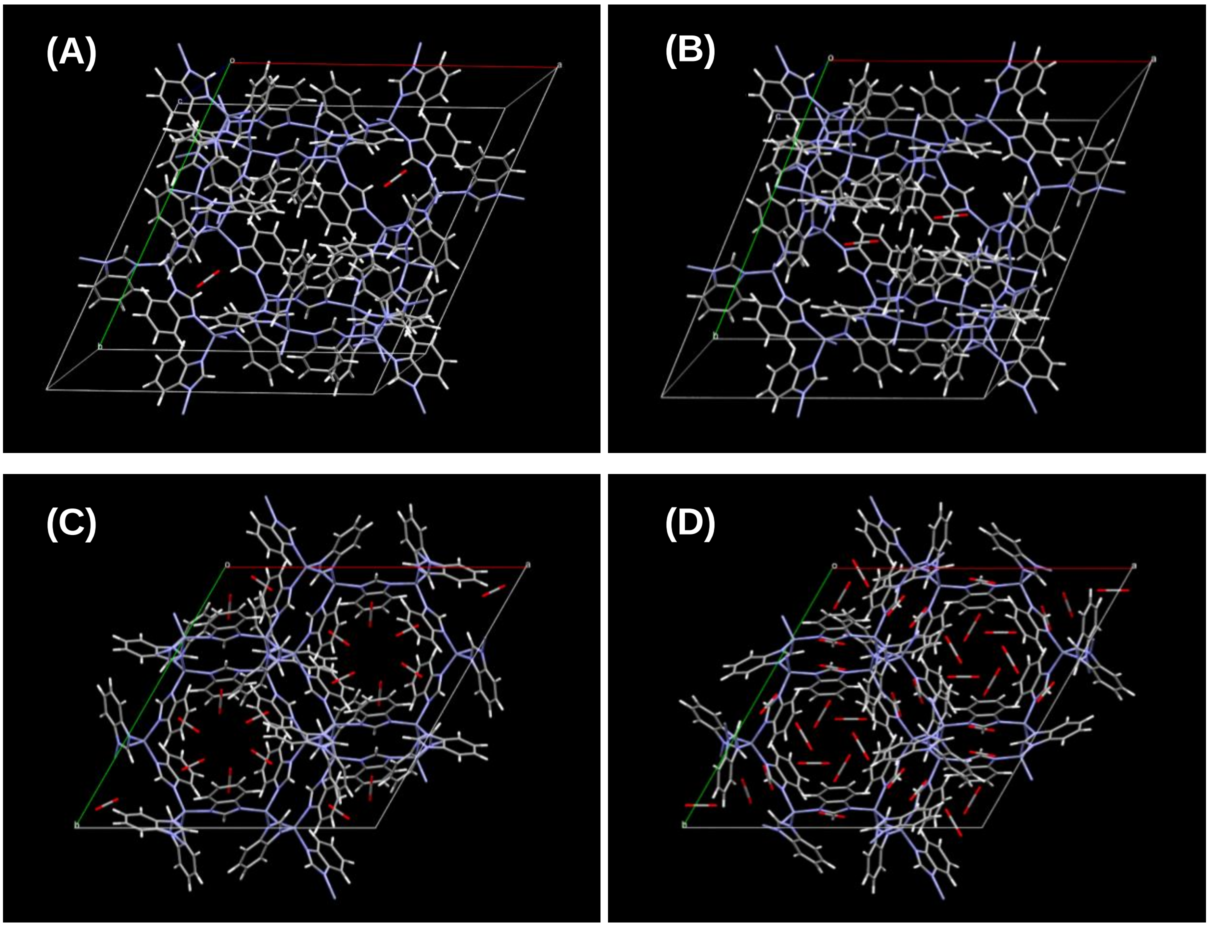

Supplementary Figure 8 Models used in DFT calculations. CO_2 is adsorbed **(A)** in pore B or **(B)** in the channel between pores A and B before the ZIF-7-II to ZIF-7-I phase transition. CO_2 is adsorbed **(C)** in pore A or **(D)** in both A and B pores after phase transition.

(A)

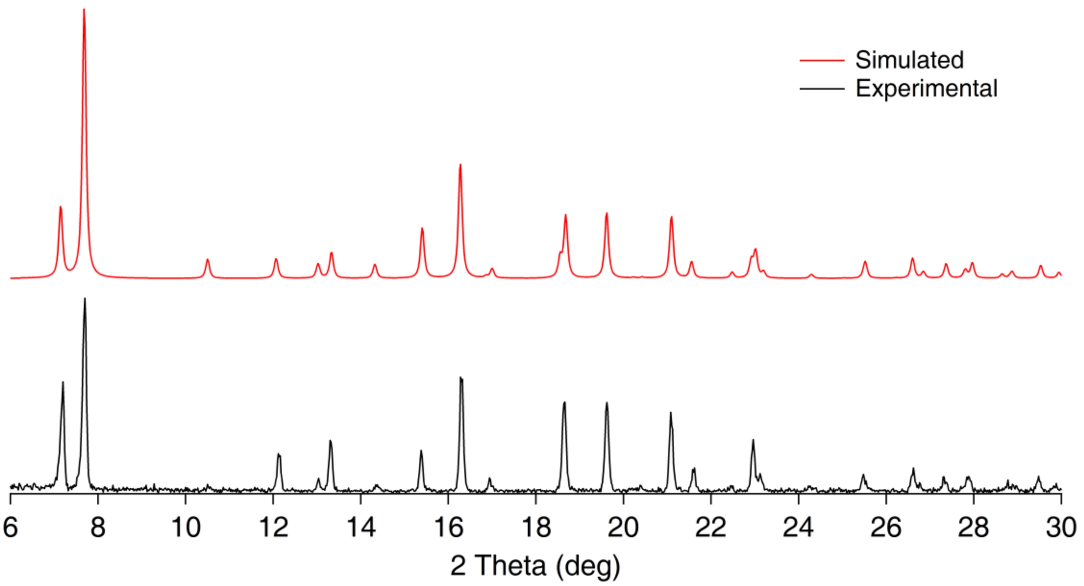

(B)

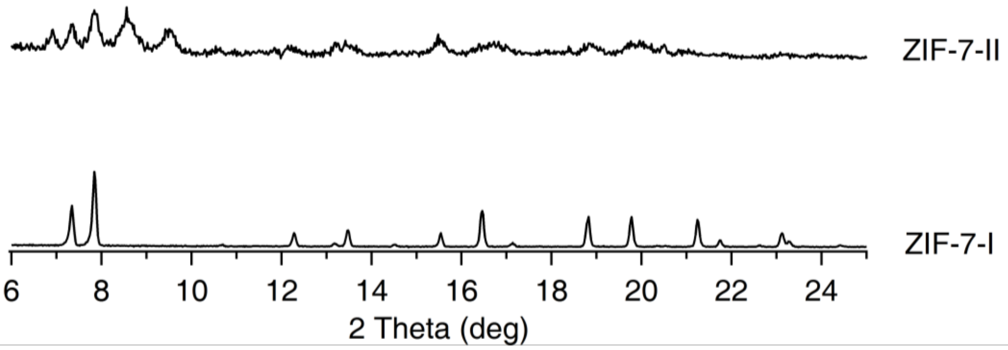

Supplementary Figure 9 (A) The XRD data of an as-synthesized ZIF-7 sample, compared with the simulated data using the ZIF-7-I crystal structure from the literature[1]. **(B)** The XRD data of ZIF-7-I and ZIF-7-II.

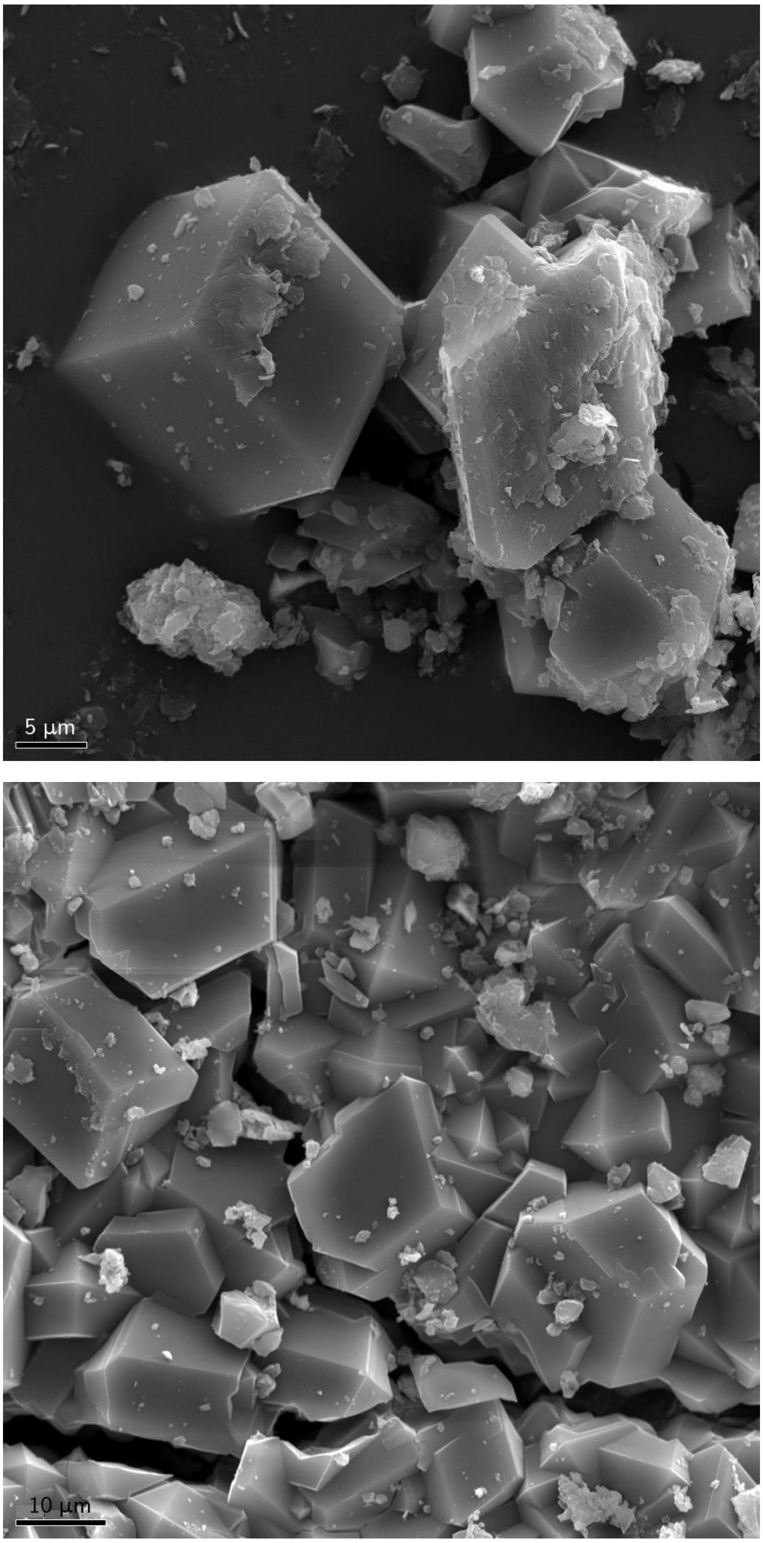

Supplementary Figure 10 The SEM images of an as-synthesized ZIF-7 sample, showing rhombohedral morphology, in line with the crystal system of ZIF-7-I phase (*R*-3). The crystal size is estimated to be around 17 μm.

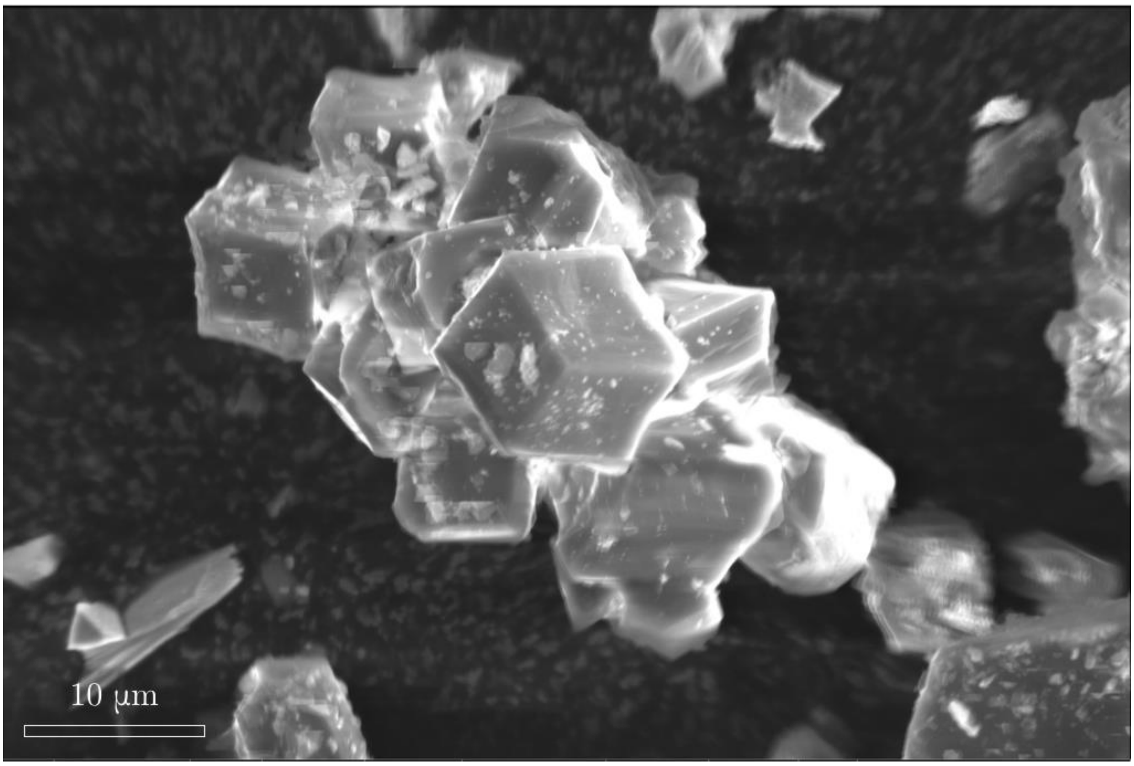

Supplementary Figure 11 The SEM image of an activated ZIF-7 sample, showing triclinic morphology, in line with the crystal system of ZIF-7-II phase (*P*-1). The crystal size is estimated to be around 7 μm. The crystal size refers to the long edge length of the crystals.

The adsorption behaviors of flexible MOFs are recently found to strongly depend on their crystal sizes. For DUT-49 and ZIF-8 mentioned in the main text, it has been demonstrated that the adsorption-induced flexibility of samples with nanometer-scaled crystal sizes is suppressed or the transition pressure is significantly higher than that for those with micrometer-scaled crystal sizes[28-31]. Our activated ZIF-7 sample contains micrometer-scaled crystals, which allows it well exhibiting adsorption-induced flexibility under our experimental conditions. The crystal sizes of all activated samples analyzed by various techniques in this manuscript are the same, so the crystal size effect is not discussed in the main text.

Supplementary Table 1 Pore size distribution of ZIF-7-I, ZIF-7-II, and ZIF-8, measured by CrystalMaker 10.3 software using their crystal structures[1,8]. The probe radius was 1.55 Å, the same as the van der Waals radius of a N_2 molecule. SOD: sodalite cage, 4M: four-member-ring pore.

ZIF-7-I

Pore center	Identity	Radius [Å]	Volume [Å^3]
(0, 0, 0)	Pore A	3.99	266
(0.33, 0.67, 0.16)	SOD	3.76	223
(0.51, 0.49, 0.06)	Pore B	3.22	140
(0.66, 0.84, 0.85)	4M	2.42	60

ZIF-7-II

Pore center	Identity	Radius [Å]	Volume [Å^3]
(0.98, 0.02, 0.50)	Pore B	4.70	436
(0.57, 0, 0.45)	Pore B	4.20	309
(0.34, 0.66, 0.20)	Pore B	3.98	264
(0, 0.52, 0.52)	Pore B	3.84	237
(0.70, 0.89, 0.87)	Pore B	3.70	212
(0.81, 0.67, 0.22)	Pore B	3.53	184
(0.53, 0.45, 0.19)	SOD	3.07	121
(0.02, 0.98, 0)	4M	2.85	97
(0.11, 0.79, 0.16)	SOD	2.77	89
(-0.02, 0.48, 0.97)	4M	2.61	74
(0.79, 0.65, 0.73)	4M	2.55	69
(0.32, 0.67, 0.63)	4M	2.52	67
(0.60, 0.62, 0.53)	Pore A	2.50	66
(0.23, 0.83, 0.91)	Pore A	2.33	53
(0.96, 0.26, 0.22)	Pore A	2.28	50
(0.51, 0, 0)	4M	2.24	47
(0.38, 0.24, 0.78)	4M	2.02	34
(0.19, 0.79, 0.59)	SOD	1.94	31

ZIF-8

Pore center	Identity	Radius [Å]	Volume [Å^3]
(0, 0, 0)	SOD	7.11	1503

Supplementary Table 2 The relationship between pressure and CO_2 uptake by ZIF-7 at 195 and 298 K.

CO_2 uptake (X, mmol·g^{-1}) is given by

$$X = \frac{m - m_0}{44 \times m_0} \times 10^3$$

Supplementary Equation 11

where m_0 is the sample weight measured under vacuum, m is the sample weight measured under CO_2.

***T* = 195 K**

p (kPa)	CO_2 uptake (mmol·g^{-1})	p (kPa)	CO_2 uptake (mmol·g^{-1})
0.0482	0.02237	31.283	4.19432
1.0821	0.91892	32.7522	4.20277
2.32	1.53877	34.2622	4.21099
3.5444	1.94028	34.956	4.21516
4.7824	2.19601	36.3028	4.22204
5.9523	2.34844	37.8129	4.23003
7.2447	2.47641	39.3093	4.23607
8.4418	2.56530	42.3702	4.24698
9.6662	2.63876	44.9958	4.25525
11.8156	2.71995	47.4173	4.26260
13.3121	2.78200	49.8932	4.27108
14.8085	2.85341	54.9267	4.28506
16.305	2.93317	59.9602	4.29735
17.815	3.07060	65.0073	4.30815
19.3795	3.31271	69.8639	4.31822
20.7943	3.52878	74.9518	4.32843
22.2907	3.79742	79.9581	4.33817
23.7736	4.06606	84.9916	4.34836
25.2836	4.14341	89.8754	4.35759
26.78	4.16542	94.9361	4.36728
28.2765	4.17736	104.3637	4.38161
29.7865	4.18617	100.3505	4.37568

***T* = 298 K**

p (kPa)	CO_2 uptake (mmol·g^{-1})	p (kPa)	CO_2 uptake (mmol·g^{-1})
-0.0607	0.00344	51.2536	0.77531
1.0549	0.01169	52.478	0.88821
2.2792	0.01870	53.7024	1.02912
3.5036	0.02510	54.9403	1.14016
4.7416	0.03096	56.1783	1.19315
5.9795	0.03666	57.3891	1.24289
7.1631	0.04221	58.5998	1.29106
8.401	0.04775	59.8106	1.32397
9.6254	0.05309	61.0485	1.35679
10.8498	0.05834	62.2865	1.37792
12.0741	0.06359	63.4972	1.40373
13.2985	0.06841	64.7352	1.42569
14.5228	0.07348	65.946	1.44353
15.7472	0.07856	67.1703	1.46212
16.9852	0.08384	68.4083	1.47728
18.2095	0.08891	69.6055	1.49410
19.4203	0.09410	70.857	1.50690
20.631	0.09937	72.0678	1.51815
21.869	0.10459	73.2785	1.53037
23.107	0.11030	74.5165	1.53964
24.3177	0.11590	75.7409	1.54739
25.5285	0.12122	76.9788	1.55363
26.7664	0.12528	78.1896	1.55984
27.9908	0.12863	79.4003	1.56596
29.2016	0.13360	80.6247	1.57170
30.4395	0.13947	81.8763	1.57658
31.6639	0.14648	83.0734	1.58113
32.8746	0.15354	84.3114	1.58628
34.1262	0.16123	85.5357	1.59037
35.337	0.16947	86.7601	1.59417
36.5477	0.17871	87.9845	1.59765
37.7993	0.18736	89.168	1.60141
39.01	0.19563	90.4468	1.60509
40.2208	0.20643	91.6439	1.60874
41.4588	0.22072	92.9091	1.61222
42.6831	0.23830	94.0927	1.61535
43.9075	0.26401	95.3306	1.61888
45.1454	0.29539	96.5414	1.62187
46.3562	0.33953	97.7929	1.62537
47.5534	0.40662	99.0445	1.62816
48.8049	0.49391	100.2281	1.63110
50.0429	0.60723	101.4524	1.63329

Supplementary Table 3 Crystallographic information of ZIF-7-I and ZIF-7-II[8].

	ZIF-7-I	ZIF-7-II
Formula	Zn(bIm)$_2$	Zn$_9$(bIm)$_{18}$
Crystal system	Rhombohedral	Triclinic
Space group	*R*-3	*P*-1
a [Å]	22.989(3)	23.948(6)
b [Å]	22.989(3)	21.354(6)
c [Å]	15.763(3)	16.349(4)
α [deg]	90	90.28(2)
β [deg]	90	93.28(2)
γ [deg]	120	108.41(1)
V [Å^3]	7214(2)	7917(3)

Supplementary Table 4 Band assignment of ZIF-7-II INS spectra.

Wavenumber (cm^{-1})	Band assignment
50–150	Zn ring deformation; linker rotation
150–215	δ N–Zn–N; ν Zn–N
230–245 (doublet)	δ2 linker at C2–C6
270–290	ν Zn–N
290–330	τ linker
415–435 (doublet)	τ Bz (raised by C3–H & C7–H wagging)
460–464	δ Zn–N–C2/6
471	δ N–C2/6–C
550	τ Bz & Im
583	δ2 Bz C4–H & C5–H (wagging)
644	τ Im
744–747	δ2 Bz C–H
771	δ1 Bz & Im
844–847	δ2 Im C1–H
895–900	δ2 Bz C3–H, C4–H & C7–H
1003–1006	δ1 Bz C–H

[1] ν: stretching; δ: bending (δ1: in-plane, δ2: out-of-plane); τ: torsion.

[2] Bz: benzene ring; Im: imidazole ring. Below shows the atom nomenclature used for the linker, H is omitted for clarity.

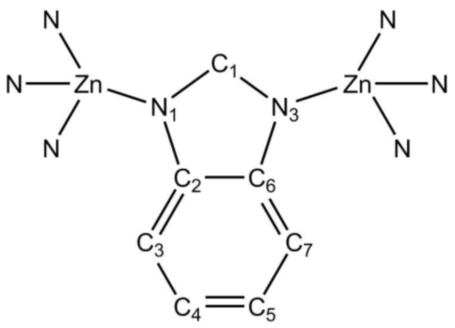

Supplementary Table 5 Force field parameters for UFF+, derived from the Universal Force Field, and used to model the framework atoms of ZIF-7-I and ZIF-7-II[16,17]. TraPPE parameters used to model CO_2 [18].

UFF+

	σ (Å)	ε (K)
C	3.431	31.270
N	3.261	20.549
H	2.571	13.103
Zn	2.462	36.928

TraPPE

	σ (Å)	ε (K)
O_{CO2}	3.05	79.0
C_{CO2}	2.80	27.0

Supplementary References

1 Park, K. S. *et al.* Exceptional chemical and thermal stability of zeolitic imidazolate frameworks. *Proc. Natl. Acad. Sci. USA* **103**, 10186–10191 (2006).

2 Thompson, S. P. *et al.* Beamline I11 at Diamond: a new instrument for high resolution powder diffraction. *Rev. Sci. Inst.* **80**, 075107–075109 (2009).

3 Thompson, S. P. *et al.* Fast X-ray powder diffraction on I11 at Diamond. *J. Synchrotron Rad.* **18**, 637–648 (2011).

4 Lu, Z. *et al.* Modulating supramolecular binding of carbon dioxide in a redox-active porous metal-organic framework. *Nat. Commun.* **8**, 14212 (2017).

5 Yang, S. *et al.* Supramolecular binding and separation of hydrocarbons within a functionalized porous metal-organic framework. *Nat. Chem.* **7**, 121–129 (2014).

6 Yang, S. *et al.* Selectivity and direct visualization of carbon dioxide and sulfur dioxide in a decorated porous host. *Nat. Chem.* **4**, 887–894 (2012).

7 Aguado, S. *et al.* Guest-induced gate-opening of a zeolite imidazolate framework. *New J. Chem.* **35**, 546–550 (2011).

8 Zhao, P. *et al.* Phase Transitions in Zeolitic Imidazolate Framework 7: The Importance of Framework Flexibility and Guest-Induced Instability. *Chem. Mater.* **26**, 1767–1769 (2014).

9 Zhao, P., Lampronti, G. I., Lloyd, G. O., Suard, E. & Redfern, S. A. T. Direct visualisation of carbon dioxide adsorption in gate-opening zeolitic imidazolate framework ZIF-7. *J. Mater. Chem. A* **2**, 620–623 (2014).

10 Perdew, J. P., Burke, K. & Ernzerhof, M. Generalized Gradient Approximation Made Simple. *Phys. Rev. Lett.* **77**, 3865–3868 (1996).

11 Kresse, G. & Furthmuller, J. Efficiency of ab-initio total energy calculations for metals and semiconductors using a plane-wave basis set. *Comput. Mater. Sci.* **6**, 15–50 (1996).

12 Kresse, G. & Furthmuller, J. Efficient iterative schemes for ab initio total-energy calculations using a plane-wave basis set. *Phys. Rev. B* **54**, 11169–11186 (1996).

13 Grimme, S. Semiempirical GGA-type density functional constructed with a long-range dispersion correction. *J. Comput. Chem.* **27**, 1787–1799 (2006).

14 Fang, H., Dove, M. T. & Refson, K. Ag–Ag dispersive interaction and physical properties of $Ag_3Co(CN)_6$. *Phys. Rev. B* **90**, 054302 (2014).

15 Dubbeldam, D., Calero, S., Ellis, D. E. & Snurr, R. Q. RASPA: molecular simulation software for adsorption and diffusion in flexible nanoporous materials. *Mol. Simul.* **42**, 81–101 (2016).

16 Rappe, A. K., Casewit, C. J., Colwell, K. S., Goddard, W. A. & Skiff, W. M. UFF, a full periodic table force field for molecular mechanics and molecular dynamics simulations. *J. Am. Chem. Soc.* **114**, 10024–10035 (1992).

17 Fairen-Jimenez, D. *et al.* Flexibility and swing effect on the adsorption of energy-related gases on ZIF-8: combined experimental and simulation study. *Dalton Trans.* **41**, 10752–10762 (2012).

18 Potoff, J. J. & Siepmann, J. I. Vapor–liquid equilibria of mixtures containing alkanes, carbon dioxide, and nitrogen. *AIChE J.* **47**, 1676–1682 (2001).

19 Reid, R. C., Prausnitz, J. M. & Poling, B. E. *The properties of gases and liquids*. (McGraw Hill Book Co., 1987).
20 Nicholson, D. & Parsonage, N. G. *Computer Simulation and the Statistical Mechanics of Adsorption*. (Academic Press, 1982).
21 Arnold, O. *et al.* Mantid—Data analysis and visualization package for neutron scattering and μSR experiments. *Nucl. Instrum. Methods Phys. Res., Sect. A* **764**, 156–166 (2014).
22 Jobic, H. in *Adsorption and Diffusion* (eds Hellmut G. Karge & Jens Weitkamp) 207–233 (Springer Berlin Heidelberg, 2008).
23 Yang, Q. *et al.* Probing the dynamics of CO_2 and CH_4 within the porous zirconium terephthalate UiO-66(Zr): a synergic combination of neutron scattering measurements and molecular simulations. *Chemistry* **17**, 8882–8889 (2011).
24 Papadopoulos, G. K., Jobic, H. & Theodorou, D. N. Transport diffusivity of N_2 and CO_2 in silicalite: coherent quasielastic neutron scattering measurements and molecular dynamics simulations. *J. Phys. Chem. B* **108**, 12748–12756 (2004).
25 Ryder, M. R. *et al.* Identifying the Role of Terahertz Vibrations in Metal-Organic Frameworks: From Gate-Opening Phenomenon to Shear-Driven Structural Destabilization. *Phys. Rev. Lett.* **113**, 215502 (2014).
26 Morsy, M. A., Al-Khaldi, M. A. & Suwaiyan, A. Normal Vibrational Mode Analysis and Assignment of Benzimidazole by ab Initio and Density Functional Calculations and Polarized Infrared and Raman Spectroscopy. *J. Phys. Chem. A* **106**, 9196–9203 (2002).
27 Cordes, M. M. & Walter, J. L. Infrared and Raman studies of heterocyclic compounds—II Infrared spectra and normal vibrations of benzimidazole and bis-(benzimidazolato)-metal complexes. *Spectrochim. Acta Part A* **24**, 1421–1435 (1968).
28 Tanaka, S. *et al.* Adsorption and Diffusion Phenomena in Crystal Size Engineered ZIF-8 MOF. *J. Phys. Chem. C* **119**, 28430–28439 (2015).
29 Zhang, C., Gee, J. A., Sholl, D. S. & Lively, R. P. Crystal-Size-Dependent Structural Transitions in Nanoporous Crystals: Adsorption-Induced Transitions in ZIF-8. *J. Phys. Chem. C* **118**, 20727–20733 (2014).
30 Krause, S. *et al.* The effect of crystallite size on pressure amplification in switchable porous solids. *Nat. Commun.* **9**, 1573 (2018).
31 Tian, T., Wharmby, M. T., Parra, J. B., Ania, C. O. & Fairen-Jimenez, D. Role of crystal size on swing-effect and adsorption induced structure transition of ZIF-8. *Dalton Trans.* **45**, 6893–6900 (2016).
