# Supplementary material for: Structural dynamics of a metal–organic framework induced by CO2 migration in its non-uniform porous structure
Source: Nat Commun. 2019 Mar 1;10:999. doi: 10.1038/s41467-019-08939-y (PMC6397191; doi:10.1038/s41467-019-08939-y)
Supplement: Supplementary file 4 — Description of Additional Supplementary Files [file 41467_2019_8939_MOESM4_ESM.docx]

Description of Additional Supplementary Files

**Supplementary Data 1:** a typical input file of DFT calculations

**Supplementary Data 2**: Input files of GCMC simulations
